# The Treatment of Contagious Ecthyma in Lambs with a Local Anaesthetic/Antiseptic Wound Formulation Lowers Serum Amyloid A Responses

**DOI:** 10.3390/ani16010017

**Published:** 2025-12-20

**Authors:** Aurora Ortín, Sergio Villanueva-Saz, Delia Lacasta, Peter Andrew Windsor, Antonio Fernández, Pablo Quilez, Hector Ruiz, Alex Gómez, David Guallar, Marta Ruiz de Arcaute

**Affiliations:** 1 Animal Pathology Department, Veterinary Faculty and Instituto Agroalimentario de Aragón-IA2 (Universidad de Zaragoza-CITA), Miguel Servet 177, 50013 Zaragoza, Spain; svs@unizar.es (S.V.-S.); dlacasta@unizar.es (D.L.); afmedica@unizar.es (A.F.); pquilez@unizar.es (P.Q.); hectorruiz353@gmail.com (H.R.); alex.gomezcalvo@gmail.com (A.G.); davidguallarabellan2002@gmail.com (D.G.); martarda@unizar.es (M.R.d.A.); 2 Ruminant Clinical Service, Veterinary Faculty, University of Zaragoza, Miguel Servet 177, 50013 Zaragoza, Spain; 3 Sydney School of Veterinary Science, The University of Sydney, Camden, NSW 2570, Australia; peter.windsor@sydney.edu.au

**Keywords:** orf virus, contagious ecthyma, lambs, local antiseptic/anaesthetic formulation, serum amyloid A

## Abstract

Contagious ecthyma (CE) is a widespread, highly contagious zoonotic skin disease of small ruminants caused by the Orf virus (ORFV), leading to substantial economic losses and welfare concerns. There is no specific treatment, with topical antiseptics and oral or parenteral antibiotics often administered for preventing secondary infections, risking antimicrobial resistance. This study assessed the effect of treating CE in lambs with an antibiotic-free topical anaesthetic/antiseptic formulation (Tri-Solfen^®^; T-S; Medical Ethics, Australia/MultiSolfen^®^; M-S; Dechra, UK). Serum amyloid A (SAA), a marker of systemic inflammation, was measured in both experimentally and naturally infected lambs allocated to treated and untreated groups. Samples were collected prior to (T0) and at 2 (T2), 7 (T7) and 14 (T14) days post-treatment in experimentally infected lambs and at T0, 10 (T10) and 20 (T20) days post-treatment in naturally affected lambs. In the experimental infection, SAA concentrations were lower in the treated group than in controls at T7 and significantly lower at T14. In the natural outbreak, SAA concentrations significantly decreased over time in the treated group, with a consistent trend toward lower values than in controls. These findings indicate that this therapeutic formulation reduces systemic inflammatory responses in lambs affected by CE, supporting its use as an alternative to antibiotics.

## Simple Summary

Contagious ecthyma (CE) is a skin disease of small ruminants with significant economic impact and welfare concerns. Vaccination is the main preventive strategy for CE, although in many countries, licenced vaccines are unavailable. Treatment typically involves antibiotics to control secondary infections, increasing the risk of antimicrobial resistance. The objective of this study was to evaluate the therapeutic effect of a non-antibiotic topical anaesthetic/antiseptic therapeutic formulation (Tri-Solfen^®^; T-S; Medical Ethics, Australia/MultiSolfen^®^; M-S; Dechra, UK) on lambs with contagious ecthyma. The concentration of a marker of systemic inflammation, serum amyloid A (SAA), was measured during the clinical phase of CE in naturally and experimentally infected lambs, in cohorts either treated or not treated with the product. In the experimental infection, the treatment modified the SAA response, peaking earlier and at lower levels than in controls and showing significantly lower values at the completion of the experimental period than in controls. In the natural outbreak, SAA levels significantly decreased over time in the treated cohort, whereas in controls, levels remained stable at high values. These results indicate that this topical formulation reduces systemic inflammation in lambs with CE, providing supportive evidence that this is a promising non-antibiotic therapeutic alternative to current practice.

## 1. Introduction

Contagious ecthyma (CE, Orf) is a highly contagious, globally distributed skin disease primarily affecting small ruminants, especially young animals in their first year of life [1]. The disease is a non-systemic, eruptive skin infection caused by the Orf virus (ORFV), a member of the family Poxviridae and genus *Parapoxvirus* [2]. Clinically, CE is characterised by papules, vesicles, pustules and proliferative scabby lesions, mainly of the muzzle and lip mucosae, often extending to the oral mucosa, nostrils, ears and eyelids and, less frequently, the feet, genitals and udder [1,3,4,5,6]. CE is a self-limiting disease, with lesions usually resolving within 6–8 weeks [4]. However, ORFV encodes immunomodulatory proteins that interfere with the host immune response, favouring reinfections and predisposing animals to secondary infections [7,8]. This virus-induced immunosuppression contributes to the risk of persistence of the infection, recurrence after live virus vaccination and secondary complications including bacterial contamination and infection [4,6]. Outbreaks of CE infection may cause substantial animal welfare concerns, with frequent antibiotic use raising the risks of antimicrobial resistance.

ORFV is mainly transmitted through skin cuts or abrasions [9]. Morbidity can reach 100%, with mortality usually <5%, although in very young animals with secondary infections, up to 90% mortality has been reported [10,11]. CE causes significant economic losses, particularly in intensive husbandry systems, due to mortality in young animals, reduced productivity linked to decreased food intake caused by painful lesions and interference in sheep marketing and trading due to clinical similarities to transboundary vesicular viral diseases [6,12,13]. As ORFV infection is also a zoonosis, humans in close contact with infected animals, particularly farmers, veterinarians, shearers and slaughterhouse workers, are occasionally infected, typically developing localised skin lesions on the hands [6,14,15].

Appropriate hygiene and disinfection measures are essential to prevent ORFV infection. The quarantine of newly introduced animals and separation of sick ones are required, as well as the thorough disinfection of facilities and materials [4]. Once ORFV enters and establishes in a flock, eradication is difficult [4]. The virus can persist in dry environments, remaining viable on various surfaces and animal by-products for long periods. Immunocompromised or persistently infected animals maintain the environmental circulation of ORFV [16].

Vaccination is the main preventive strategy for CE, although in many countries, licenced vaccines are unavailable for sheep and goats [6,17,18]. Existing vaccines include scab-based and attenuated tissue culture vaccines [8,19,20], although these may revert to virulence. Their use is not recommended on farms without a history of CE, as they can contaminate the environment [4]. Despite these issues, live CE vaccines are used widely in Australia, where they successfully suppress the disease in many flocks [6,19]. As the duration of protective immunity from current vaccines is variable, the development of a safe, more effective and readily accessible ORFV vaccine remains a priority [1,17]. Promising prototypes of DNA and subunit vaccines targeting ORFV B2L and ORFV F1L proteins have been described [21,22,23,24], and current research is focusing on genetic manipulation to delete ORFV virulence genes and generate attenuated vaccines with stronger immunoprotective properties [25,26].

As there is no specific therapy for CE, current approaches include standard hygiene practices, local topical antiseptics and topical or parenteral antibiotics, administered mainly to manage the risk of secondary infections. Local antiseptics reported include sodium permanganate and salicylic acid [27], potassium permanganate and boric acid ointments [9], 3% iodine solution, gentian violet [28] and hypochlorous acid [29]. Traditional herbal remedies including sesame or castor oil, giant milkweed juice and *Euphorbia* have also been used [4]. Although ineffective against ORFV [30], antibiotics applied topically or systemically are widely used to treat secondary bacterial infections, contributing to the risks of antimicrobial resistance (AMR) [13,31]. With the increasing global concerns of AMR from livestock including in small ruminant medicine, there is a need to explore alternatives to antibiotic therapy for CE.

Tri-Solfen^®^ (T-S, Medical Ethics, Melbourne, Australia), later registered and marketed in Europe as MultiSolfen^®^ (M-S; Dechra, Northwich, UK), is a wound therapeutic formulation combining two local anaesthetics (lidocaine and bupivacaine), adrenaline and an antiseptic (cetrimide) in a gel matrix. Developed in Australia for surgical husbandry procedures in sheep, it creates a barrier effect, blocking nociception, reducing pain and accelerating healing [32]. It was later shown to be effective in treating oral mucosal lesions in cattle with foot and mouth disease [33,34]. Its low pH (~2.7) may also confer virucidal activity, potentially reducing ORFV expression and transmission. Preliminary trials in CE-affected lambs suggested reduced viral loads in treated animals, although no differences in clinical lesion progression was observed [18]. In experimentally infected lambs, T-S treatment did not affect weight gain or lesion progression, possibly due to the deep epithelial location of the induced-CE lesions and to an inadequate treatment protocol administered too early and with too few applications [31]. However, in a later field study in a commercial flock, when the product had already been registered in Europe, repeated M-S treatment applied after vesicle eruption and on three occasions reduced the number and severity of lesions in treated versus control animals, suggested as evidence of efficacy and supporting its use in field conditions [29].

Acute phase proteins (APPs) are a large family of unrelated proteins [35] with plasma concentrations that may increase (positive APPs) or decrease (negative APPs) in response to inflammation, infection, trauma or stress [36,37]. Synthesised mainly in the liver, APPs contribute to limiting microbial growth, minimising tissue damage and restoring homeostasis [38]. Their measurement has diagnostic, prognostic and monitoring applications in infectious and inflammatory conditions, as well as in subclinical disease detection [37,39]. The APP profile is species-specific; in ruminants, haptoglobin (Hp) and serum amyloid A (SAA) are the most relevant, showing up to 1000-fold increases in response to stimuli [36,40,41]. However, the SAA response in lambs is generally stronger than that of Hp [36]. Hp levels remain unchanged in lambs following aversive stimuli, including tail docking and castration, whereas this APP is an effective marker for monitoring these processes in cattle [40]. Our group also has evidence of a stronger SAA response than that of Hp in feedlot lambs [42]. In lambs, SAA shows a particularly rapid and intense response, with peaks within 24–48 h after stimulus and normalisation within 4–7 days if no further challenge occurs [36,43].

This study evaluated the evolution of the SAA response during the clinical phase of CE in naturally and experimentally infected lambs, in cohorts either treated or not treated with M-S/T-S. The objective was to assess whether the potential therapeutic effect of this formulation on CE-affected lambs could also be reflected in the serum levels of the most relevant APP in lambs.

## 2. Materials and Methods

All procedures were supervised and approved by the Ethics Committee for Animal Experiments of the University of Zaragoza (reference PI33/21). Animal care and use complied with the Spanish Policy for Animal Protection (RD53/2013), which aligns with the European Union Directive 2010/63 on the protection of animals used for experimental and other scientific purposes.

Two experimental studies were conducted: one with lambs experimentally infected with ORFV and another on a commercial sheep farm naturally affected by a CE outbreak. In both studies, animals were allocated into two cohorts: (i) treated with M-S or T-S or (ii) untreated (control). SAA was measured in treated and untreated lambs throughout the experimental period.

### 2.1. Study in Lambs Experimentally Infected with ORFV

The experimental design previously described [31] for evaluating the effect of T-S treatment in lambs experimentally infected with ORFV was followed in this study. Briefly, 50 healthy, four-day-old male lambs were recruited from a Lacaune dairy sheep farm that had not experienced CE outbreaks in the previous three years. All lambs were tested by PCR and ELISA and confirmed negative for ORFV before being transferred to the facilities of the Veterinary Faculty of Zaragoza. Once there, they were randomly allocated into two independent and fully isolated pens, with 25 lambs in each.

All lambs were experimentally infected with aliquots of 1 mL of inoculum containing 10^4^ TCID_50_ (median tissue culture infectious dose) of ORFV. The inoculum was administered using an intradermal inoculation gun, distributed across the lips, gums and palate [31]. Briefly, an average of 17 inoculations were administered for each lamb (1.7 mL/animal), split between the right, left and central upper lips; right, left and central lower lips; and the upper and lower aspects of the interior of the mouth (gums and/or palate). Eight days after infection, once the first CE lesions appeared, lambs from pen 2 (treatment group) were treated with T-S (Tri-Solfen^®^, Medical Ethics, Melbourne, Australia), spraying 1.5 mL of the product with a dosing gun over the lips and inside the mouth, ensuring the complete coverage of all lesions. Treatment was repeated three days later (11 days post-inoculation) in the affected areas of the face (Table 1). Lambs from pen 1 (control group) did not receive any treatment.

For this study, blood samples without anticoagulant were collected by jugular venipuncture from lambs in both groups at four time points: the day before the first treatment (T0) and at 2 (T2), 7 (T7) and 14 (T14) days after the first treatment (Table 1). Sera were obtained, aliquoted and stored at –20 °C until the analysis of SAA concentrations.

### 2.2. Study in Lambs Naturally Affected by Contagious Ecthyma

This study was part of a larger experiment that evaluated the effect of M-S treatment in a commercial sheep farm affected by a CE outbreak [29]. Briefly, in that experiment, 150 Lacaune neonatal lambs aged 25–30 days were selected on the basis of clinical presentation with skin or oral lesions consistent with CE, and infection was confirmed by PCR. The lambs were randomly divided into three cohorts (groups C, D and E) of 50 animals each. Lambs in group C were treated with M-S (Dechra, Northwich, UK), spraying 1.5 mL of the product with a dosing gun over the lips and inside the mouth, ensuring the complete coverage of all lesions. The treatment was applied on three occasions with 3-day intervals (Table 2). Animals in group D were treated daily with hypochlorous acid using the same technique as in group C, with lambs in group E serving as untreated controls.

For the present study, 12 lambs from group C (M-S treated) and 12 lambs from group E (control) were randomly selected. Blood samples without anticoagulant were collected by jugular venipuncture at three time points: the day before the first treatment (T0) and at 10 (T10) and 20 (T20) days after the first treatment (Table 2). Samples were processed to obtain sera, aliquoted and stored at –20 °C until the measurement of SAA concentrations.

### 2.3. Serum Amyloid A (SAA) Concentration

SAA concentrations were measured using a solid-phase sandwich ELISA kit (PHASE™ Serum Amyloid A Assay, Tridelta Development Ltd., Maynooth, Ireland) following the manufacturer’s instructions. Absorbance was read with a Multiskan MS microplate reader (Labsystems, Helsinki, Finland). Sample concentrations were calculated by interpolation from the calibration curve generated with the kit calibrators, adjusting for the corresponding dilution factor.

All samples were analysed in duplicate, and the mean value was used. Samples with absorbance values outside the calibration range were re-analysed after further dilution to bring them within range. The intra- and inter-assay coefficients of variation (CVs) were 5.0% and 11.4%, respectively. The assay sensitivity was 0.3 μg/mL.

### 2.4. Statistical Analysis of Results

Data were analysed using IBM SPSS Statistics version 26.0 (Armonk, NY, USA). Non-parametric tests were applied, as the variable did not meet normality assumptions. The Friedman test was used to evaluate the effect of time on SAA concentrations. Differences between T-S/M-S-treated and untreated groups at each time point were assessed with the Mann–Whitney test. In all analyses, differences were considered statistically significant at *p* < 0.05.

## 3. Results

### 3.1. Study in Lambs Experimentally Infected with ORFV

**Figure 1:**
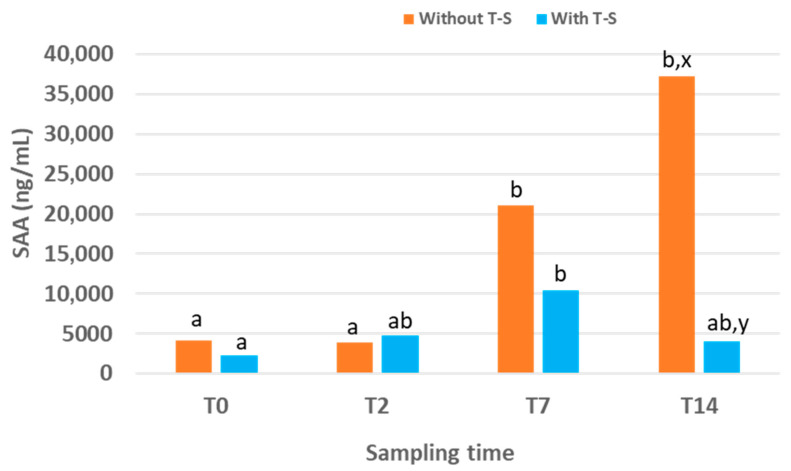
Serum amyloid A (SAA) concentration over time in lambs experimentally infected with ORFV that were treated or not treated (n = 25 for each group) with Tri-Solfen^®^ (T-S). Samples were collected at T0 (prior to treatment), T2 (2 days), T7 (7 days) and T14 (14 days) after the first treatment. Different letters (a,b) indicate significant differences between different sampling times. Different letters (x,y) indicate significant differences between groups.

The evolution of SAA concentrations in lambs experimentally infected with ORFV is displayed (Figure 1), comparing the group treated with T-S and the untreated control group. The median values together with the first and third quartiles for each sampling time point and the percentage of reduction in SAA in treated lambs compared to untreated ones are also displayed (Table 3).

In the control group, SAA levels increased over time, reaching the highest value, 37,283 (6166.51–154,459.75) ng/mL, at the end of the experimental period (T14). At this time point, SAA concentrations were significantly higher than at T2 and T0 (*p* = 0.030 and *p* = 0.013, respectively). No significant differences were observed between T0 and T2, although the value at T7 was significantly higher than those at both T0 (*p* = 0.024) and T2 (*p* = 0.020).

In the group treated with T-S, SAA levels also increased over time but only during the first half of the experimental period. The peak occurred at T7, with a value of 10,475 (2410–24,240.7) ng/mL, which was lower than the peak recorded in the control group at T14. In the treated group, SAA concentration at T7 was higher than that at T0, T2 and T14, although significant differences were only detected between T7 and T0 (*p* = 0.001). No significant differences were found between groups at T0 or T2. However, SAA concentrations at T7 and T14 were lower in the T-S-treated group compared with the control group (they were reduced by 50.29% and 89.11%, respectively), with the difference reaching significance at T14 (*p* = 0.041).

### 3.2. Study in Lambs Naturally Affected by Contagious Ecthyma

**Figure 2:**
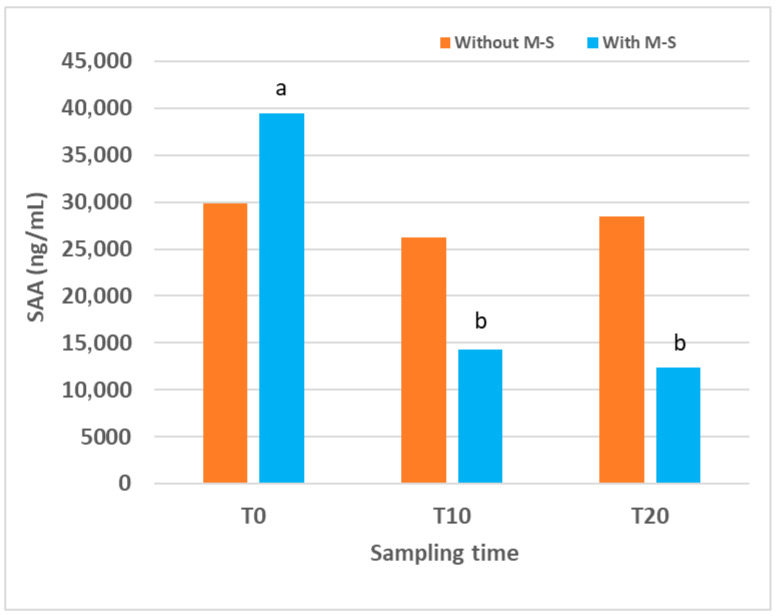
Serum amyloid A (SAA) concentrations over time in lambs naturally affected by contagious ecthyma, treated or not treated (n = 12 for each group) with Multi-Solfen^®^ (M-S). Samples were collected prior to treatment (T0) and at 10 (T10) and 20 (T20) days after the first treatment. Different letters (a,b) indicate significant differences between different sampling times.

The evolution of SAA concentrations in lambs naturally affected by CE is shown (Figure 2), comparing the group treated with M-S and the untreated control group. The median values together with the first and third quartiles for each sampling time point and the percentage of reduction in SAA in treated lambs compared to untreated ones are also displayed (Table 4).

In the control group, SAA concentration at the beginning of this study was very high, 29,843 (16,205.50–111,879.62) ng/mL, and remained similar at all sampling times, with no significant differences detected over time. In the group of lambs treated with M-S, SAA concentration at T0 was also very high, 39,456 (30,996.17–105,669.65) ng/mL, but decreased after treatment. Values at T10 and T20 were significantly lower than that at T0 (*p* = 0.030 for both). When comparing the two groups, no significant differences were detected at any sampling time. However, a trend towards lower values was observed in the M-S-treated group compared with controls at T10 (*p* = 0.061) and T20 (*p* = 0.081), and SAA levels were reduced by 45% and 56.75% at T10 and T20, respectively.

## 4. Discussion

CE is a globally distributed, highly contagious zoonotic viral skin disease, causing significant economic losses in affected sheep and goat farms. Only a few registered vaccines exist, and these are not readily available in many European and Asian countries.

There is currently no effective treatment for CE, and local antiseptics and antibiotics are frequently used, increasing the risk of AMR.

T-S/M-S has been proposed as a potential non-antibiotic therapeutic alternative for this disease. In an initial study with 50 experimentally infected lambs, T-S treatment did not affect the clinical progression of CE lesions, a result attributed to an inadequate treatment protocol administered too early and with too few applications [31]. By contrast, in a subsequent field study in a commercial flock affected by a CE outbreak, M-S was applied after vesicle eruption and on three occasions, resulting in fewer lambs with ORFV-associated lesions and milder lesions compared with untreated animals. This finding suggested that topical M-S can be effective under field conditions if the appropriate therapeutic protocol is applied [29].

The present study aimed to provide further evidence of the therapeutic effect of T-S/M-S in CE-affected lambs. SAA, the most relevant APP in lambs, was analysed in blood samples collected from lambs participating in two previously published studies [29,31].

Our finding in the experimentally infected lambs was that T-S treatment modified the SAA response, peaking earlier and at lower levels than in controls. Whilst SAA levels in the control group increased progressively, reaching the maximum at the end of the experimental period (T14), in the group treated with T-S, the peak occurred earlier, at T7, followed by a decline. Consequently, at T14, treated lambs had significantly lower SAA concentrations than controls. Although it was previously reported that there were no clinical differences between groups [31], our SAA data from this study suggest a potential therapeutic effect of T-S on the inflammatory response in CE experimentally induced lambs, highlighting the importance of optimising treatment protocols to enable the product to achieve its full potential.

In support of this aim, in the published field study in which M-S was applied after vesicle eruption in naturally infected lambs and on three occasions [29], clinical differences between groups were reported. The present analysis of SAA concentrations in a subsample of animals from that study provides additional evidence for the therapeutic potential of M-S in CE under field conditions. In naturally affected lambs, baseline SAA concentrations at T0 were already very high, reflecting the fact that CE lesions were more advanced at the time of the first sampling than in the experimental trial. In this group, M-S treatment was associated with a significant decrease in SAA values at T10 and T20 compared with T0, whereas in controls, levels remained stable at high values. Although intergroup comparisons did not reach statistical significance, a consistent trend towards lower SAA concentrations was observed in treated lambs at both later time points, suggesting that the sample size may not have been sufficient to detect differences with greater statistical power. These results indicate that M-S treatment may reduce the systemic SAA response, consistent with the therapeutic suppression of the inflammatory response, in naturally affected treated lambs as well, in agreement with the clinical findings.

Our results of a reduced SAA response following T-S/M-S therapy are consistent with those observed in lambs treated with T-S following tail docking [43]. In both of our studies, SAA concentrations were much higher than the baseline values reported for lambs in the literature [44,45], although this was expected, since SAA increases markedly in response to inflammation, infection or trauma. Elevated T0 values were also observed in both studies, likely reflecting the fact that CE lesions were developing or had already appeared and triggered the systemic response prior to the first sampling. The SAA response typically rises within 24–48 h after the triggering event [38]. However, this initial increase was much more pronounced in naturally affected lambs, likely due to more advanced lesion development at T0 compared with the experimental study.

In both studies, SAA concentrations did not return to baseline during the observation period, despite reports in the literature describing a rapid normalisation within 4–7 days if no further stimuli occur [36,43]. In CE, lesions may persist for 6–8 weeks [4,5], possibly due to recurrent reinfections [1,4] or, more likely, the severe pathological nature of the proliferative dermatitis in CE lesions, characterised by the ballooning of the cytoplasm of hyperplastic basal epithelial cells deeply embedded in the vicinity of the dermis, which is likely to be ongoing and refractory to rapid healing [6]. Although T-S/M-S has demonstrated virucidal activity and a capacity to reduce viral load in CE lesions [18], the penetration of actives into the deeper basal epithelium may be insufficient to be curative, enabling ongoing infection to sustain the SAA response. Multiple-dose regimens and longer treatment periods may therefore be necessary, potentially extending for at least 4 weeks, the time usually required for lesion resolution.

## 5. Conclusions

Although T-S/M-S cannot be expected to fully eliminate ORFV from proliferative basal epidermal lesions, our findings suggest that it is a promising therapy for CE, capable of reducing inflammation, improving animal welfare and helping control secondary infections without promoting AMR. Further, our results also support the potential of SAA as a biomarker for monitoring acute phase responses during CE and for assessing disease progression. These findings should encourage further research into optimising CE treatment protocols and to validate SAA as a practical tool for monitoring field outbreaks.

## Figures and Tables

**Table 1:** Treatment and sampling schedule in lambs experimentally infected with Orf virus.

			Days Post-Treatment				
	−8 EI ^†^	−1	0	2	3	7	14
Severity of the lesions			Initial lesions				
T-S treatment ^§^			1T-S		2T-S		
Blood sampling ^‡^		T0		T2		T7	T14

^†^ EI = Experimental infection. ^§^ T-S = Tri-Solfen^®^; 1T-S = first dose of treatment; 2T-S = second dose of treatment. Control group animals remained untreated. ^‡^ Blood samples were collected from treated and untreated lambs prior to treatment (T0) and 2 days (T2), 7 days (T7) and 14 days (T14) after the application of the first dose of treatment.

**Table 2:** Treatment and sampling schedule in lambs naturally affected by contagious ecthyma.

		Days Post-Treatment				
	−1	0	4	8	10	20
Severity of the lesions		A range of skin and oral lesions				
M-S treatment ^§^		1M-S	2M-S	3M-S		
Blood sampling ^‡^	T0				T10	T20

^§^ M-S = Multi-Solfen^®^; 1M-S = first dose of treatment; 2M-S = second dose of treatment; 3M-S = third dose of treatment. Control group animals remained untreated. ^‡^ Blood samples were collected from treated and untreated lambs prior to treatment (T0) and 10 days (T10) and 20 days (T20) after the application of the first dose of treatment.

**Table 3:** Median values and 1st and 3rd quartiles of serum amyloid A (SAA) concentrations at different sampling times in lambs experimentally infected with ORFV, treated or not treated with Tri-Solfen^®^ (T-S), and percentage of reduction in SAA in treated lambs compared to untreated ones.

			SAA (ng/mL)		
Sampling Time ^§^	Group ^†^ n = 25	Median	1st Quartile	3rd Quartile	% of Reduction
T0	T-S	2297.56	1045.20	4836.25	
	C	4107.55	1320.02	9028.42	
T2	T-S	4747.10	2610.00	10,649.00	---
	C	3837.47	558.23	15,721.45	
T7	T-S	10,475.45	2410.00	24,240.7	50.29
	C	21,075.05	3377.95	77,487.62	
T14	T-S	4058.22	2046.45	27,375.00	89.11
	C	37,283.77	6166.51	154,459.75	

^§^ T0 = prior to treatment; 2 days (T2), 7 days (T7) and 14 days (T14) after the application of the first dose of treatment. ^†^ T-S = group of lambs treated with Tri-Solfen^®^; C = control.

**Table 4:** Median values and 1st and 3rd quartiles of serum amyloid A (SAA) concentrations at different sampling times in lambs naturally affected by contagious ecthyma, treated or not treated with Multi-Solfen^®^ (M-S), and percentage of reduction in SAA in treated lambs compared to untreated ones.

			SAA (ng/mL)		
Sampling Time ^§^	Group ^†^ n = 12	Median	1st Quartile	3rd Quartile	% of Reduction
T0	M-S	39,456.50	30,996.17	105,669.65	
	C	29,843.00	16,205.50	111,879.62	
T10	M-S	14,300.30	5726.02	22,225.20	45
	C	26,269.5	16,551.75	45,727.95	
T20	M-S	12,326.00	2799.02	23,381.50	56.75
	C	28,499.00	8699.99	71,160.02	

^§^ T0 = prior to treatment; 10 days (T10) and 20 days (T20) after the application of the first dose of treatment. ^†^ M-C = group of lambs treated with Multi-Solfen^®^; C = control group (untreated lambs).

## Data Availability

The raw data supporting the conclusions of this article will be made available by the authors on request.
